# Psychological stress induces an increase in cholinergic enteric neuromuscular pathways mediated by glucocorticoid receptors

**DOI:** 10.3389/fnins.2023.1100473

**Published:** 2023-02-14

**Authors:** Justine Blin, Camille Gautier, Philippe Aubert, Tony Durand, Thibauld Oullier, Laetitia Aymeric, Philippe Naveilhan, Damien Masson, Michel Neunlist, Kalyane Bach-Ngohou

**Affiliations:** ^1^Nantes Université, CHU Nantes, INSERM, The Enteric Nervous System in Gut and Brain Disorders, IMAD, Nantes, France; ^2^Nantes Université, CHU Nantes, Department of Biochemistry, Nantes, France; ^3^Université d’Angers, Department of Biology, Angers, France

**Keywords:** enteric nervous system, stress, glucocorticoid receptor, acetylcholine, motility

## Abstract

**Introduction:**

Repeated acute stress (RASt) is known to be associated with gastrointestinal dysfunctions. However, the mechanisms underlying these effects have not yet been fully understood. While glucocorticoids are clearly identified as stress hormones, their involvement in RASt-induced gut dysfunctions remains unclear, as does the function of glucocorticoid receptors (GR). The aim of our study was to evaluate the involvement of GR on RASt-induced changes in gut motility, particularly through the enteric nervous system (ENS).

**Methods:**

Using a murine water avoidance stress (WAS) model, we characterized the impact of RASt upon the ENS phenotype and colonic motility. We then evaluated the expression of glucocorticoid receptors in the ENS and their functional impact upon RASt-induced changes in ENS phenotype and motor response.

**Results:**

We showed that GR were expressed in myenteric neurons in the distal colon under basal conditions, and that RASt enhanced their nuclear translocation. RASt increased the proportion of ChAT-immunoreactive neurons, the tissue concentration of acetylcholine and enhanced cholinergic neuromuscular transmission as compared to controls. Finally, we showed that a GR-specific antagonist (CORT108297) prevented the increase of acetylcholine colonic tissue level and *in vivo* colonic motility.

**Discussion:**

Our study suggests that RASt-induced functional changes in motility are, at least partly, due to a GR-dependent enhanced cholinergic component in the ENS.

## Introduction

Repeated acute stress (RASt) is known to be associated with gastrointestinal (GI) disorders or dysfunctions such as visceral hypersensitivity and altered gastrointestinal motility, both of which lead to decreased gastric emptying and enhanced colonic transit time, or altered epithelial functions, such as increased epithelial secretion or permeability (Bhatia and Tandon, 2005; Reed et al., 2016; Meerveld and Johnson, 2018). However, the mechanisms underlying RASt-induced changes in gut functions have not yet been fully decrypted (Bonaz et al., 2002; Bhatia and Tandon, 2005; Meerveld and Johnson, 2018), especially the mediators and receptors involved.

Repeated acute stress is associated with the activation of every component of stress response. This response can be two-fold: on the one hand, stimulation of the sympathetic nervous system induces a rapid release of neuromediators such as catecholamines with adrenaline and noradrenaline, leading to rapid synaptic effects (Mittal et al., 2017). The activation of the hypothalamic-pituitary-adrenal (HPA) axis, on the other hand, induces slower effects and potentially longer-term changes (Datson, 2008). Activation of the HPA axis is characterized by secretion of corticotropin-releasing hormone (CRH) by the hypothalamus, which induces the release of adreno-corticotropic hormone (ACTH) by the pituitary gland, and finally the secretion of glucocorticoids (GC) by the adrenal glands (Larauche et al., 2009; [Bibr B45][Bibr B15]; Taché, 2015). CRH has been the most widely studied of the mediators released following HPA activation and involved in RASt effects upon GI functions. In particular, CRH modulates GI functions both through the central nervous system (CNS) (Martínez et al., 2004; Tsukamoto et al., 2006) and the autonomic nervous system, in which both the sympathetic nervous system and the enteric nervous system (ENS) may be involved (Taché and Bonaz, 2007; Taché et al., 2009; Gourcerol et al., 2011).

Corticotropin-releasing hormone, in particular, can directly act on the neurons of the ENS, an integrative neuronal network located all along the gastrointestinal tract, involved in the regulation of gastrointestinal functions such as motility and secretion (Neunlist et al., 2013; Furness et al., 2014; Spencer and Hu, 2020). CRH effects upon the ENS were shown to be mediated in a region and function-dependent manner by the activation of two receptors sub-types, corticotropin-releasing factor receptor 1 and 2 (CRF-R1 and CRF-R2). Numerous studies have tested the hypothesis that stress could impact digestive function by acting on these receptors (Rivier et al., 2003). Furthermore, the activation of each of these two receptors by CRH leads to functionally distinct responses: while activation of CRF-R1 increases colonic motor response, activation of CRF-R2 inhibits gastric emptying (Martínez et al., 2004). In addition, activation of CRF-R2 by CRH increases mucosal chloride secretion in the colon *via* enteric pathways (Liu et al., 2021).

When compared to CRH, the role of GC in RASt-mediated effects upon GI functions and its putative impact upon the ENS remains largely unknown. GC effects are mediated *via* two major nuclear receptors, i.e., the mineralocorticoid receptor (MR) and the glucocorticoid receptor (GR). GC have a high affinity for MR receptors, which are often activated under basal conditions. In contrast, GC have a lower affinity for GR receptors which are activated under “inducible” conditions, such as following HPA activation (Datson, 2008; Revollo and Cidlowski, 2009; Oakley and Cidlowski, 2013). GC activation of GR results in nuclear translocation of cytoplasmic GR and binding to the GR response element, leading to various transcriptional processes. The effects of corticosterone are limited by its degrading enzymes 11-β hydroxysteroid deshydrogenase type 2 (11βHSD-2) (Chapman et al., 2013).

Glucocorticoid receptor expression has been identified in other neuronal systems besides the ENS such as the sympathetic system, more specifically in dorsal root sensory neurons or in specific brain structures (Lerch et al., 2017). In CNS and in dorsal root sensory neurons, activation of GR by GC has been shown to regulate neuromediators synthesis or different neuronal processes, such as plasticity and sprouting (Lerch et al., 2017; Buurstede et al., 2022). In the ENS, corticosterone has been shown to modulate calcium concentration in enteric neurons and increase the proportion of choline acetyltransferase (ChAT) in rat primary ENS culture (Lowette et al., 2014; Aubert et al., 2019b). However, although RASt has been shown to increase colonic motility *via* CRF1 receptors, its ability to regulate ENS and colonic motility *via* GR remains unknown.

In this context, our study aimed to determine whether the ENS expresses GR receptors and whether RASt-induced effects on ENS phenotype and on functional remodeling are mediated, at least in part, *via* GR-dependent pathways.

## Materials and methods

### Animals

Our study was carried out in accordance with French standard ethical guidelines for laboratory animals (Agreement 02376.01) as well as with ARRIVE guidelines.^1^ All experiments involving mice were approved by the ethics committee for animal experimentation of Pays de la Loire (Apafis 6751) based at the Nantes University CHU, of which our INSERM unit is a part. Adult male C57BL/6 mice (5–6 weeks; Janvier Labs, Le Genest-Saint-Isle, France) weighing 25–30 g were used. Mice were housed in cages with free access to food and tap water. Animals were quarantined under controlled conditions in terms of illumination (12 h light/dark cycle), temperature, and humidity.

### Water avoidance stress (WAS) protocol

**FIGURE 1:**
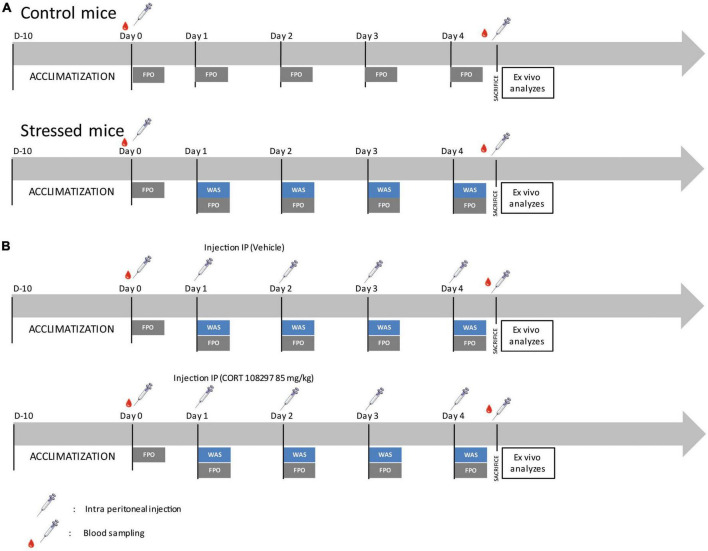
Experimental design. **(A)** Schematic timeline depicting the experimental design applied to control and water avoidance stress (WAS) mice for the assessment of *in vivo* and *ex vivo* colonic motility. FPO, fecal pellet output. **(B)** Schematic timeline depicting the experimental design applied to mice treated with CORT 108297 (85 mg/kg) or vehicle (sesame oil) for assessment of *in vivo* and *ex vivo* colonic motility.

For stress exposure, a single 1 h WAS between 8 and 9 a.m. was performed daily when the mice were 5–6 weeks old for 4 consecutive days, corresponding to the repeated acute stress protocol (Figure 1A). Mice were placed on an elevated circular platform that was placed 1 cm above the water level (10 cm height; 4 cm diameter) and positioned at the center of a plastic tank (52 × 37 × 27 cm^3^) filled with water at room temperature. This protocol was adapted from a previous study (Reed et al., 2016).

### Pharmacological treatment protocol

The specific GR antagonist, CORT108297 (kindly provided by Dr. Azel Hunt, Corcept, Drive Menlo Park, CA, USA) or the vehicle (sesame oil, Millipore Sigma, Burlington, MA, USA) were injected intraperitoneally at a dose of 85 mg/kg 1 h before the WAS procedure (Figure 1B). This dose was selected following a literature review (Zalachoras et al., 2013; Beaudry et al., 2014; Meyer et al., 2014; Pineau et al., 2016).

### *In vivo* measurement of colonic motility

Following the procedures described in previous studies (Tasselli et al., 2013; Rincel et al., 2019), to assess the fecal pellet output (FPO), control mice were placed individually in clean cages without bedding, food, or water for fecal pellet collection during 1 h. For mice subjected to WAS, the fecal pellet collection was performed for 1 h during the WAS time period while the mice were on the platform. Fecal pellets were collected and counted immediately after expulsion (Figure 1).

### *Ex vivo* measurement of colonic motility

As previously described (Aubert et al., 2019a), at the end of the fecal pellet output collection from both the control mice and the mice after the WAS treatment, on the fourth day, both the control and stressed animals were killed by cervical dislocation. Tissues were immediately transferred to cold HBSS (Eurobio, Courtaboeuf, France) after surgical resection and brought to the laboratory. Strips of longitudinal muscle were dissected and placed into an organ chamber (Radnoti, CA, USA) with 15 mL of Krebs solution at 37°C, continuously bubbled with 95% O_2_ and 5% CO_2_. The contractile response of muscle strip was continuously recorded using isometric force transducers (No. TRI202PAD, Panlab, Cornellã, Spain) coupled to a computer equipped with the PowerLab 8/30 System and the Labchart data analysis software (AD Instruments, Spechbach, Germany). Strips were stretched with an initial tension of 1 g and reached a mean tension of 0.7 g (range 0.5–0.9) after an equilibrium period of 60 min. Next, strips were subjected to electrical field stimulation (EFS) using a STG 4008 MCS electrical stimulator (Multi Channel Systems, Reutlingen, Germany). EFS parameters were as follows: train duration, 10 s; pulse frequency, 20 Hz; pulse duration, 400 μs; pulse amplitude 11 V. This procedure was repeated 3 times with a space of 4 min between repetitions. The following drugs were then added sequentially to the baths after a 10-min incubation period: nitro-L-arginine methyl ester (L-Name, 50 μM; Sigma-Aldrich, Merck, Darmstadt, Germany), an inhibitor of the nitric oxide synthase (NOS); and then atropine (1 μM; Sigma-Aldrich, Merck, Darmstadt, Germany), a muscarinic receptor antagonist. The same EFS stimulation protocol was repeated. Next, tissues were washed 5 times over a 10-min period and then allowed to recover for an additional 20 min. At the end of each experiment, a dose-response curve was created by measuring the area under the curve of the bethanechol-induced contraction. Bethanechol is a selective muscarinic receptor agonist (Millipore Sigma, Burlington, MA, USA). The response was measured for 2 min after bethanechol (10^–9^–10^–2^ M) was added. All values were normalized to the tissue weight.

### Blood sampling and corticosterone assessment

Mice were bled from their tail, at day 0 (D0) and at day 4 (D4), at the end of the 1 h-long WAS test (as described in Figure 1). Samples were obtained by quickly clipping the distal tip of the tail with a razor blade and collecting ∼50 μL of blood into EDTA-containing tubes.

In order to minimize hormonal variability due to circadian fluctuations, all procedures were performed during the circadian cycle of the diurnal corticosterone rhythm, before 11:00 a.m. After collection, blood samples were centrifuged at 1007 g, for 15 min at 4°C and then plasma samples were kept at −20°C. Corticosterone plasma levels were measured by enzymatic immunoassay according to the manufacturer’s instructions (Laboratoire IDS, Pouilly-en-Auxois, France).

### Acetylcholine assay

After the mice were killed, portions of their distal colons were removed and lysed in a RIPA buffer (Merck Millipore, Fontenay-sous-Bois, France) containing phosphatase inhibitor cocktail III (Sigma-Aldrich, Merck, Darmstadt, Germany) and protease inhibitors cocktail (Roche, Neuilly-sur-Seine, France). Briefly, tissues were crushed in the RIPA buffer using a “Precellys 24” tissue homogenizer (Bertin technologies, St Quentin-en-Yvelines, France), followed by sonication with a “Vibra-Cell 75 186” device (Sonics, Newton, CT, USA). The amount of proteins was assessed with a Bradford reagent using a BioPhotometer D30 spectrophotometer (Eppendorf, Montesson, France). Acetylcholine (ACh) concentration was determined in tissue homogenates using Amplex Red, acetylcholine/acetylcholinesterase assay kit (Invitrogen Thermofisher Scientific, Waltham, MA, USA) and normalized to the protein amount.

### Western blot analysis

As previously described (Prigent et al., 2019), protein extraction from colonic tissues was performed with NucleoSpin RNA/Protein Kit (Macherey-Nagel, Hoerdt, France, Cat# 740966) according to the manufacturer instructions. Samples were further prepared for electrophoresis by diluting with a NuPAGE sample buffer (Life Technologies, Saint-Aubin, France, Cat# NP0008) then heated at 98°C for 5 min. Lysates were separated using the NuPAGE 4–12% Bis-Tris gels (Life Technologies, Cat# NP0336BOX) together with the 2-(*N*-morpholino)ethanesulfonic acid/sodium dodecyl sulfate running buffer (Life Technologies, Cat# IB23002) before electrophoretic transfer to nitrocellulose membranes (Life Technologies, Cat# NP0002) with the iBlot2 Dry Blotting System (Life Technologies, Cat# IB21001). Membranes were then blocked for 1 h at 21°C in Tris-buffered saline (Sigma, Cat# T5912) with 0.1% (v/v) Tween-20 (Sigma, Cat# P1379) and 5% (w/v) non-fat dry milk and incubated overnight at 4°C with the following primary antibodies: rabbit anti-GR (D8H2, 3660S 1:500, Cell Signaling, Danvers, MA, USA), mouse monoclonal anti-β-actin (1:10000; Sigma, Cat#A5441, RRID:AB_476744). Bound antibodies were detected with horseradish peroxidase-conjugated anti-rabbit (Life Technologies Cat# 31460, diluted 1:5000) or anti-mouse antibodies (Sigma, Cat# A9044, diluted 1:5000) and visualized by enhanced chemiluminescent detection (Biorad, Clarity ECL, Marnes-la-Coquette, France, Cat# 170-5061).

### Quantitative PCR analysis

As previously described (Schemann et al., 1993), 1 μg of purified mRNA by Nucleospin RNA/Protein kit was denatured and converted to cDNA using the SuperScript III Reverse Transcriptase (Life Technologies). qPCR was performed using a StepOnePlus RealTime PCR Instrument (Life Technologies) with a FastSYBR Green Master Mix kit (Applied Biosystems, Foster City, CA, USA). Ribosomal protein S6 (RPS6) transcript was used as a reference. The relative expression of the gene of interest was measured by the 2^–ΔΔCt^ method. The following primers were used:

• NR3C1 # NM_008173, forward: 5′-GCAGTGGAAGGACAGC ACAAT-3′; reverse: 5′-CGTTTTTCGAGCTTCCAGGTTC-3′
• RPS6 forward: 5′-GAAGCGCAAGTCTGTTCGTG-3′; Reverse: 5′-GTCCTGGGCTTCTTACCTTCT-3′

### Immunofluorescence staining

Distal colon segments were fixed in 0.1M PBS containing 4% paraformaldehyde at room temperature for 3 h. Whole mounts of longitudinal muscle and myenteric plexus were obtained by microdissection and permeabilized with PBS containing 10% horse serum (HS) and 4% Triton X-100 for 2 h at room temperature. Tissues were then incubated with the following primary antibodies: rabbit anti-ChAT (1:1000, a gift from Professor M. Schemann, Hannover, Germany) (Schemann et al., 1993), mouse anti-neuronal NOS (nNOS; 610308, 1:500; BD Biosciences), rabbit anti-GR (D8H2, 3660S 1:500, Cell Signaling), human anti-Hu (Bodin et al., 2021) (gift from the CHU of Nantes; 1:5000) diluted in PBS containing 10% horse serum and 0.5% Triton X-100 for 24 or 48 h at room temperature. After washing, tissues were incubated for 2 h at room temperature with the appropriate secondary antibodies, respectively, anti-rabbit CY5 (1:500), anti-rabbit CY3 (1:500) and anti-human FP488 (1:200), and mounted Glycerol 60% (vol/vol) (Thermo Fisher Scientific). Nuclei were stained with 4′ 6*-*Diamidino*-*2*-*phenylindole dihydrochloride (DAPI D9542; 1:10000; Sigma Aldrich, Paris, France).

### Image analysis and quantification

Images from immunostained tissues were acquired with a digital camera (Axiozoom, Carl Zeiss, Jena, Germany). The number of Hu-, ChAT-, nNOS- and GR-neurons was counted in 20 ganglia per animal. The data were expressed as the percentage of ChAT-, nNOS-, or GR-neurons normalized to the total number of Hu-neurons.

For GR immunostaining, confocal microscopy was performed using a Nikon A1R confocal inverted microscope (Nikon France SAS, Champigny sur Marne, France) with a Nikon X60 Plan-Apo numerical aperture (NA) 1.4 oil-immersion objective (MicroPICell core facility).

The intensity of cytoplasmic and nuclear expression of GR receptor was analyzed in 10 ganglia for all Hu identified neurons. Image analysis was performed with the ImageJ (ROI Manager) following immunohistochemical staining for GR, Hu, and DAPI. First, neurons were identified using Hu and its perimeter defined manually using ImageJ. Next, the neuronal nucleus (identified with DAPI staining) perimeter was defined using ImageJ. Then both neuronal perimeter and nucleus perimeter were overlaid on the GR staining. Finally, the intensity of GR staining in the neuronal nucleus and in the neuronal cytoplasm were calculated using ImageJ. The intensity of neuronal cytoplasmic GR abundance was determined by subtracting the intensity of the GR abundance in the nucleus from that of the whole neuron. Finally, the ratio of nuclear over cytoplasmic GR abundance was calculated. This analysis was blinded to the treatment group.

### Statistical analyses

Statistical analyses were performed using GraphPad Prism 5 (GraphPad Software, San Diego, CA, USA). Data were represented as means ± SD. Group comparisons were made using the Mann–Whitney *U* test or by 2-way ANOVA and Dunnett’s multiple comparisons test as indicated. Values of *p* < 0.05 were considered statistically significant. When necessary we tested our data with the Grubbs test to detect Outliers. Only one outlier has been identified in this study (see Figure 5C) and has been removed, leaving 15 points in the control group against 16 in the WAS group.

All data generated or analyzed during this study are included in this published article and its Supplementary material.

## Results

### Enteric neurons of mice distal colonic ENS express the GR glucocorticoid receptor

**FIGURE 2:**
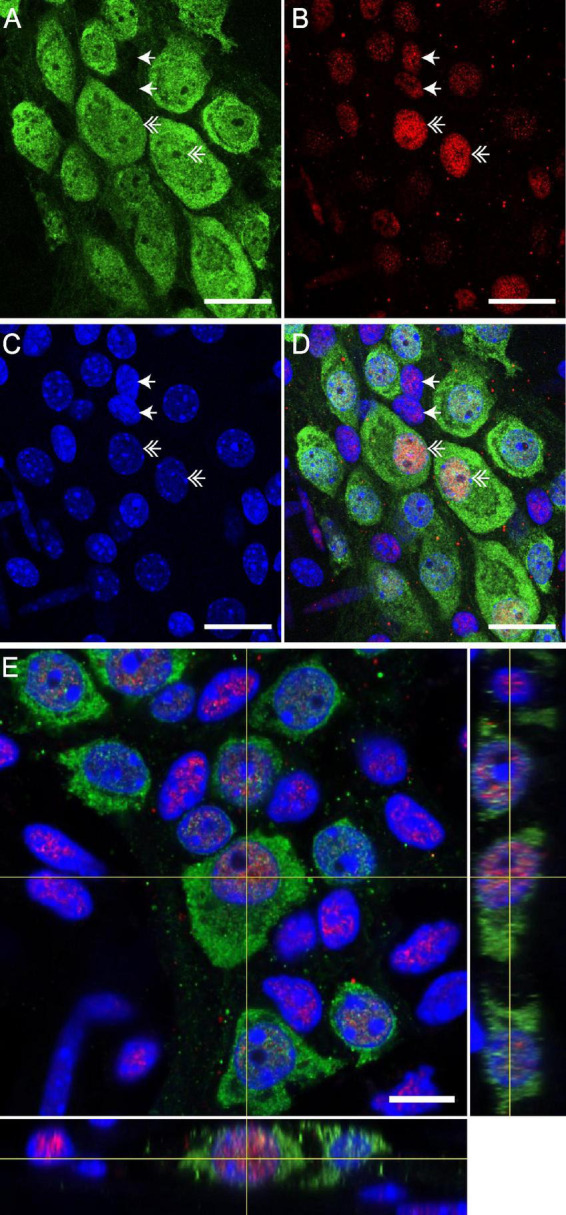
GR is expressed by neurons of enteric nervous system (ENS) in distal colon of mice. Confocal acquisition of double immunolabeling for Hu-IR **(A)** and GR **(B)** in the myenteric plexus of the distal colon in the mice. 4′,6-diamidino-2-phenylindole (DAPI) was used for immunolabelling of nuclei **(C)**. The merged image **(D)** revealed the colocalization of GR with the DAPI labeling, suggesting a nuclear localization of GR. Scale bars of panels **(A–D)** represent 25 μm. Double arrows show GR expression by Hu-IR enteric neurons and simple arrows show GR expression by non-neuronal cells. **(E)** Z-reconstruction of confocal micrographs of myenteric plexus after double immunostaining with Hu and GR antibodies showing the preferential nuclear localization of GR in the neuron. Scale bars of panel **(E)** represent 10 μm.

We first aimed to determine whether enteric neurons express GR receptors. We therefore performed GR immunohistochemical labeling in whole mounts of longitudinal muscle-myenteric plexus of distal colon segments. Neurons were stained using antibodies directed against neuronal Hu-IR protein. Using high resolution confocal microscopy, we showed that GR is expressed in a large proportion of Hu-immunoreactive (IR) enteric neurons (Figures 2A–D). We focused on the Hu-IR cells and did not study other enteric cell types. For these Hu-IR cells, we observed that GR was preferentially located in the nucleus using the orthogonal viewing mode (Figure 2E). GR expression was also reported in other non-neuronal cells within and outside enteric ganglia.

### Stress increases nuclear translocation of GR

**FIGURE 3:**
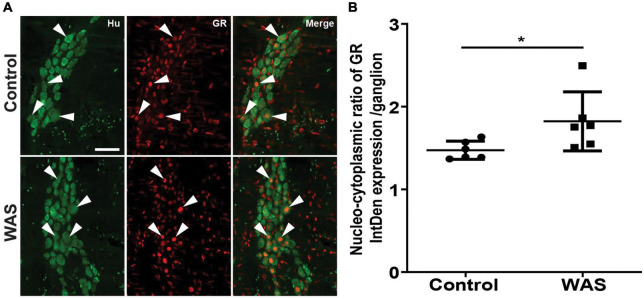
Stress increases the nuclear translocation of GR in neurons of the myenteric plexus. **(A)** Double immunolabeling for Hu (green; arrowheads indicate neuron markers) and GR (red; arrows indicate glucocorticoid receptor markers in neurons) in the distal colons of the myenteric plexus in control and WAS mice. A scale bar represents 25 μm. **(B)** Quantitative nucleo-cytoplasmic ratio of GR labeling intensity (IntDen) per ganglion. Data are represented as means ± SD (*n* = 6/group) *p < 0.05 (Mann–Whitney *U* test).

We next aimed to determine whether RASt modulates GR expression in enteric neurons. We first showed that repeated acute WAS significantly increased serum corticosterone levels as compared to control mice (282.6 ± 113.9 ng/ml vs. 51.8 ± 19.2, respectively; *p* < 0.0001) (Supplementary Figure 1). Next, we showed that the intensity of GR expression was not modified by RASt (Supplementary Figure 2) in Hu-IR cells. Furthermore, western blot and PCR analysis of colonic tissue showed that RASt did not modify protein and mRNA expression of GR as compared to control (Supplementary Figure 3). However, using immunohistochemistry we were able to show that the ratio of the intensity of nuclear GR abundance over cytoplasmic GR abundance was significantly increased by RASt as compared to control (Figure 3).

### RASt-induced phenotypic modulation of ENS neurons is mediated by GR in mice distal colon

**FIGURE 4:**
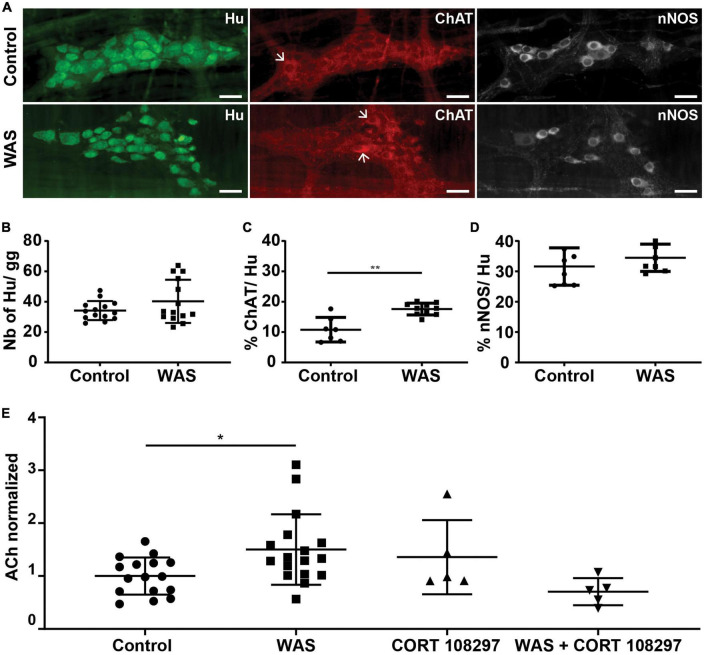
Stress induces modifications of enteric neuronal phenotype. **(A)** Immunolabeling for Hu, ChAT, and nNOS in the myenteric plexus of the distal colon of mice in control or WAS conditions (representative images are shown). Scale bars represent 30 μm. Arrows point out ChAT-positive neurons. **(B)** Quantitative analysis for the neurons per ganglion (gg) in control and WAS groups. Data are represented as means ± SD (*n* = 14/group) *p* = 0.511 (Mann–Whitney *U* test). **(C)** Proportion of neurons expressing ChAT (ChAT/Hu) in control and WAS groups. Data are represented as means ± SD (*n* = 7 for control mice and *n* = 10 for WAS mice) **p < 0.01 (Mann–Whitney *U* test). **(D)** Proportion of neurons expressing nNOS (nNOS/Hu) in control and WAS groups. Data are represented as means ± SD (*n* = 8/group) *p* = 0.328 (Mann–Whitney *U* test). **(E)** Distal colonic acetylcholine tissue levels (ACh) in control and WAS conditions ± GR antagonist CORT108297 (μmol/μg of protein). Data are presented as means ± SD. One way ANOVA test and Dunnett’s multiple comparisons test was used for panel **(E)**. *p < 0.05.

We next aimed to determine whether RASt modulates the expression of key enzymes such as ChAT and nNOS which are involved, respectively, in the synthesis of ACh and NO, two key neuromodulators involved in the regulation of gut motility (Figure 4A). We first showed that repeated acute WAS did not modify the number of neurons (identified by Hu-IR) as compared to control (Figure 4B). Interestingly, repeated acute WAS induced a significant 63% increase in the proportion of ChAT-IR neurons as compared to control (Figure 4C). However, RASt did not modify the proportion of nNOS-IR neurons (Figure 4D). We next showed that the increase in the proportion of ChAT-IR neurons was associated with a significant increase in ACh concentration in colonic tissue as compared to control (Figure 4E). Interestingly, RASt-induced increase in ACh colonic tissue level was prevented by the pretreatment of mice with CORT108297 (85 mg/kg), the selective GR receptor antagonist, prior to RASt.

### RASt increases colonic motility in a GR-dependent manner

Then, we asked whether RASt-induced changes in ENS phenotype were associated with changes in colonic motor functions in a GR-dependent manner.

**FIGURE 5:**
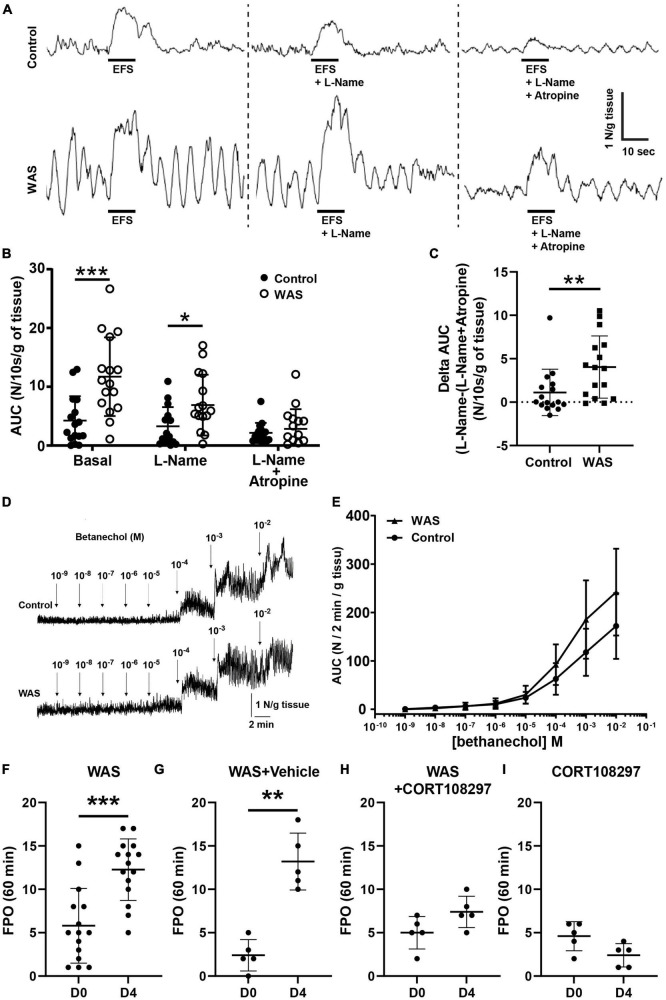
WAS mice exhibit increased colonic motility. Distal colonic longitudinal muscle segments were stimulated by electrical field stimulation (EFS). **(A)** Representative traces of EFS contractile response in basal conditions, with L-Name and with L-Name + Atropine in control and WAS mice. **(B)** Mean EFS-induced area under curve (AUC) WAS of distal colon longitudinal muscle segment after a treatment with L-Name or L-Name + Atropine (*n* = 16/group). **(C)** Amplitude of atropine-sensitive EFS-induced AUC in control and in WAS mice (*n* = 16/group). **(D)** Representative trace responses to increasing doses of bethanechol of isolated colon from control and WAS mice. **(E)** Dose–response AUC contractions of distal colonic longitudinal muscular segments from control and WAS mice to increasing doses of bethanechol (*n* = 16/group). **(F)** *In vivo* propulsive motor function of the colon assessed by measuring FPO of mice after 1 h of WAS treatment at day 0 (D0) before RASt and on day 4 (D4), after 4 days of repeated WAS (*n* = 16/group). **(G)** Comparison of the *in vivo* propulsive motor function for mice receiving the vehicle (sesame oil), between day 0 and day 4, after 4 days of repeated WAS (*n* = 5/group). **(H)** Comparison of the *in vivo* propulsive motor function for mice receiving the CORT108297, between day 0 and day 4, after 4 days of repeated WAS (*n* = 5/group). **(I)** Comparison of the *in vivo* propulsive motor function for mice receiving the CORT108297, between day 0 and day 4, without any WAS stimulation (*n* = 5/group) (*p*-value = 0.07). Data are represented using bars as means ± SD *p < 0.05, **p < 0.01, and ***p < 0.001 Mann–Whitney *U* test were used for all panels, except panel **(E)** where one-way ANOVA was used.

We analyzed *ex vivo*, in an organ chamber, the neuromuscular response of segments of the distal colon to electrical field stimulation. First, we analyzed the spontaneous contractile motility pattern in the colon. We did not find any difference of mean basal tension or amplitude of contractions from control or WAS mice. In contrast, WAS significantly increased the basal area under the curve (AUC) of the spontaneous contractile activity (Supplementary Figure 4). We next analyzed the EFS-induced contractile responses were analyzed in the absence or presence of L-NAME and/or atropine (Figure 5A). The EFS-induced AUC was larger in WAS mice than in controls (n = 16; p < 0.001) (Figure 5B). In the presence of L-NAME, EFS-induced AUC was still significantly larger in WAS as compared to controls (*n* = 16; *p* = 0.026) (Figure 5B). Finally, in the presence of atropine, EFS-induced AUC were similar in both WAS and control mice (*n* = 16; *p* = 0.56) (Figure 5B). However, the amplitude of the atropine-sensitive AUC component was higher in the WAS group as compared to controls (*n* = 16; *p* = 0.005) (Figure 5C). Of interest, the amplitude of the atropine-sensitive AUC of the spontaneous contractile activity was significantly higher in the WAS group as compared to controls (*n* = 8; *p* < 0.001) (Supplementary Figure 4).

In order to exclude the possibility that the differential response to atropine between WAS and control was reflecting a differential sensitivity of cholinergic receptors on muscles, we measured the muscle response to cholinergic muscarinic agonist (bethanechol) stimulation. We showed that bethanechol induced a similar dose-dependent increase in contractile response in tissues of both control and WAS mice (Figures 5D, E).

Finally, we aimed to determine whether changes in neuromuscular transmission induced by WAS in the distal colon were associated with functional changes *in vivo*. We showed that FPO were significantly higher in WAS mice as compared to control, with, respectively, 5.8 ± 4.3 in control mice vs. 12.3 ± 3.5 in WAS mice (*p* < 0.001; *n* = 16) (Figure 5F). Interestingly, we showed that pretreatment of WAS mice with CORT108297 (85 mg/kg) prevented WAS increase in FPO (Figures 5G, H). Basal administration of CORT108297 did not modify FPO (Figure 5I).

## Discussion

Our study demonstrates that myenteric neurons express the glucocorticoid receptor GR in the distal colon. We further showed that RASt enhanced the nuclear translocation of GR in myenteric neurons and induced an increase in the following: the number of ChAT-IR neurons, the ACh tissue level in the distal colon, and cholinergic neuromuscular transmission. We also showed that RASt caused an increase in motility and in the distal colonic ACh level, both of which were prevented by the GR specific antagonist CORT108297. Altogether, our study suggests that RASt-induced functional changes in colonic motility are, at least partly, due to GR-dependent ENS cholinergic up-regulation. Our findings could, therefore, open novel therapeutic perspectives by targeting GR expressed by colonic neurons in gut functional disorders associated with an altered HPA axis.

A first major finding of our study is to consider corticosterone as an important regulator of ENS and gut functions in response to RASt. Indeed, until recently, a RASt-induced increase in CRH was considered to mediate the majority of stress-induced effects upon gut functions (Rivier et al., 2003; Taché et al., 2005; Fukudo, 2007), in part *via* its activation of CRH receptors expressed by enteric neurons. However, an increasing amount of evidence suggests that corticosterone could directly modulate neuronal functions in the ENS. First, corticosterone can be produced in the ENS micro-environment. Indeed, 11-β hydroxy steroid deshydrogenase type 1 (11-βHSD1), the bidirectional enzyme converting inactive 11-dehydrocorticosterone to active corticosterone, has been identified in populations of enteric neurons (Lowette et al., 2014). However, in the ENS, the role of 11-βHSD1 remains unknown. Secondly, concerning the direct effects of corticosterone on enteric neurons, acute corticosterone application onto myenteric neurons has been shown to increase intracellular calcium while long term (20 h) incubation of ENS culture with corticosterone reduced EFS induced Ca^2+^ response in neurons (Lowette et al., 2014). In addition, the incubation of primary culture of ENS with corticosterone increased the proportion of ChAT-IR neurons (Aubert et al., 2019b), echoing the findings we obtained *in vivo* following RASt. Interestingly, increased enteric cholinergic activity was also reported in a porcine model of early weaning stress which represents a psychological model of HPA axis activation (Medland et al., 2016).

Another important finding of our study is the identification of GR in RASt-induced effects upon ENS and GI functions. Expression of GR in the gut has been previously reported in various cells of the GI tract, i.e., epithelial and immune cells of the lamina propria (Zheng et al., 2013; Zong et al., 2019). GR is also expressed in neuronal cell types of the peripheral nervous system such as neurons of the dorsal root ganglia and also in region-specific parts of the brain (Lerch et al., 2017). In the enteric neurons, GR expression has been recently revealed by single-cell sequencing by Zeisel et al. (2018). We have now demonstrated its expression in myenteric neurons by immunohistochemistry. In neurons of the CNS, GC/GR interactions are associated with morphological plasticity and alterations of neuronal functions (Madalena and Lerch, 2016). However, GR-induced neuronal changes vary with stress and cell type (Madalena and Lerch, 2017). For instance, acute exposure of dorsal root sensory neurons to GC were associated with increased synaptic plasticity while more chronic exposure is associated with dendritic atrophy (Madalena and Lerch, 2016, 2017). Whether region-specific expression of GR and/or cell-type specific expression of GR also exist in the ENS remains currently unknown. Following RASt we observed an increase in the ratio of nuclear/cytoplasmic abundance of GR in enteric neurons. This finding is consistent with the nuclear translocation of GR upon its binding with its ligand, leading to activation of GR-dependent transcriptomic response (Meijer et al., 2019). As a consequence of GR activation by RASt, our study demonstrated an increase in the proportion of ChAT-IR neurons associated with an increase in ACh tissue level. In addition, we showed that RASt increased both cholinergic spontaneous contractile response and enhanced EFS-induced contractile responses, as assessed by the sensitivity of these response to atropine, supporting the functional impact of the increased cholinergic phenotype induced by RASt. In contrast, RASt did not modify NO dependent neuromuscular responses consistent with no change in proportion of nNOS-IR neurons. However, one cannot exclude the contribution of an inducible NO (iNOS) component in RASt effects upon motility (Traini et al., 2021) have shown that WAS increased iNOS expression in rat myenteric neurons. Although it was well-known that acute stress could activate cholinergic myenteric neurons (Miampamba et al., 2007), no study had identified the ability of GC to modulate ENS functions and an associated impact upon colonic motility. While CRH-mediated effects can be considered a short-term response leading to acute activation of enteric neurons (Hanami and Wood, 1992; Tache et al., 2018), GC could preferentially induce long-term changes in neurons *via* GR (Datson, 2008).

The mechanisms underlying the increased proportion of cholinergic neurons and colonic tissue ACh content induced by RASt have not been directly addressed in our study. However, it is tempting to speculate that following WAS, activation of GR increases the level of ChAT mRNA which would result in an increased abundance of ChAT protein in enteric neurons. On the one hand, this would lead to an increased detection in the number of ChAT-IR neurons detected by immunohistochemical methods (as ChAT antibodies have a relatively low sensibility). This increase, in turn, would explain the higher proportion of ChAT-IR neurons following WAS as compared to control observed in our study (as no change in the total number of Hu-IR neurons was reported following WAS). On the other hand, increased ChAT protein abundance in enteric neurons would lead to an increased synthesis of ACh and contribute to the increased colonic tissue content of ACh we observed. The hypothesized increase level of ChAT mRNA could be due either to increased transcriptional activity of ChAT Promoter or the stabilization of ChAT mRNA. In the CNS, some studies suggest that GC could indeed indirectly activate ChAT expression by modulating activity of neurotrophic factors like nerve growth factor (Shi et al., 1998; Gonzalez et al., 1999; Borges et al., 2013). Another pathway potentially involved in GR-induced modulation of cholinergic phenotype is the ability of GC to down-regulate NF-KB expression in cellular models of neurons (Toliver-Kinsky et al., 2000). Since NF-KB acts as a repressor of ChAT transcription (Toliver-Kinsky et al., 2000), GR-induced NF-kB inhibition could ultimately lead to increased ChAT expression. Therefore, future specific studies would be warranted to explore mechanisms underlying GR-induced changes in cholinergic expression in the ENS.

Finally, our study demonstrated the efficacy of a GR-specific antagonist at preventing neuromediator and functional remodeling induced by RASt. Indeed, pretreatment with CORT108297 decreased FPO and ACh colonic tissue level in mice subjected to WAS. CORT108297 is a GR antagonist with a strong affinity (Meyer et al., 2014), and, unlike the classic GR antagonist RU486, has the advantage of being devoid of a progesterone receptor inhibitory effect (Clark, 2008; Sindelar et al., 2014). CORT108297 has been shown to be an antagonist specific to GR at the dose used in this study (Zalachoras et al., 2013; Beaudry et al., 2014; Meyer et al., 2014; Pineau et al., 2016), and to potently suppress corticosterone responses to a forced swim test (FST) and restraint stress (Solomon et al., 2014).

Altogether, our data suggest that corticosterone represents a novel actor involved in the regulation of ENS and gut functions. Better understating mechanisms involved in ENS regulation by corticosterone in health and disease might lead to the development of novel therapeutic approaches targeting GR or downstream targets to improve GI dysfunctions associated with HPA dysfunctions.

## Data availability statement

The raw data supporting the conclusions of this article will be made available by the authors, without undue reservation.

## Ethics statement

The animal study was reviewed and approved by French standard ethical guidelines for laboratory animals (Agreement 02376.01).

## Author contributions

MN and KB-N conceived and designed the study. JB, CG, PA, TO, and TD conceived and conducted the experiment(s). PN performed the immunohistochemical analysis. MN, KB-N, JB, and CG analyzed the results. JB, KB-N, PN, and MN wrote the manuscript. DM, PA, TD, and LA critically reviewed the manuscript. All authors reviewed the manuscript and approved the submitted version.

## Footnotes

1
https://arriveguidelines.org
