# Supplementary material for: Psychological stress induces an increase in cholinergic enteric neuromuscular pathways mediated by glucocorticoid receptors
Source: Front Neurosci. 2023 Feb 14;17:1100473. doi: 10.3389/fnins.2023.1100473 (PMC9971731; doi:10.3389/fnins.2023.1100473)

**Psychological stress induces an increase in cholinergic enteric neuromuscular pathways mediated by glucocorticoid receptors.**

**Supplemental data S1 - RASt increases serum corticosterone in WAS mice**

Corticosterone assessment by immuno-enzymatic assay. Data are represented as means +/- SD (n= 16/ group) **** p<0.0001 (Mann-Whitney U test).

**
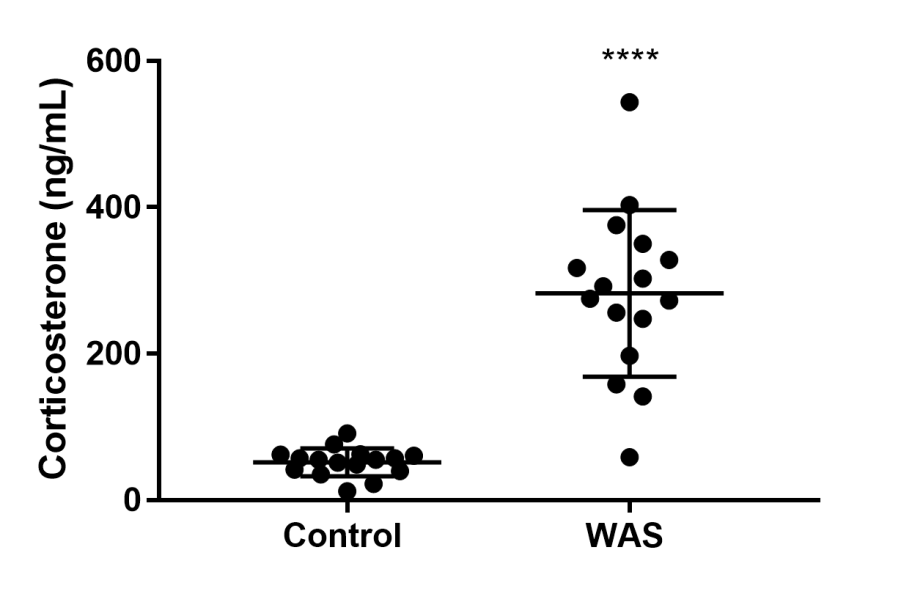
**

**Supplemental data S2 – RASt do not modify the intensity of GR expression in Hu-IR cells**

Quantitative analysis of GR labeling intensity (IntDen) per ganglion in the nucleus and in the cytoplasm of HU-IR cells. Data are represented as means +/- SD (n= 6/ group). No significant difference (Mann-Whitney U test).

**Supplemental data S3 - RASt did not modify protein and mRNA expression of GR as compared to control**

(a) Quantitative analysis of GR protein expression in distal colonic biopsies of WAS and control mice. Note that GR and β-actine were performed on the same nitrocellulose membrane. (b) Quantitative analysis of GR mRNA expression in distal colonic biopsies of WAS and control mice. Data are represented as means +/- SD (n= 8/ group). No significant difference. Original and unprocessed versions of blot after GR revelation (c) and after actine revelation (d). Original and unprocessed version of agarose gel (e).


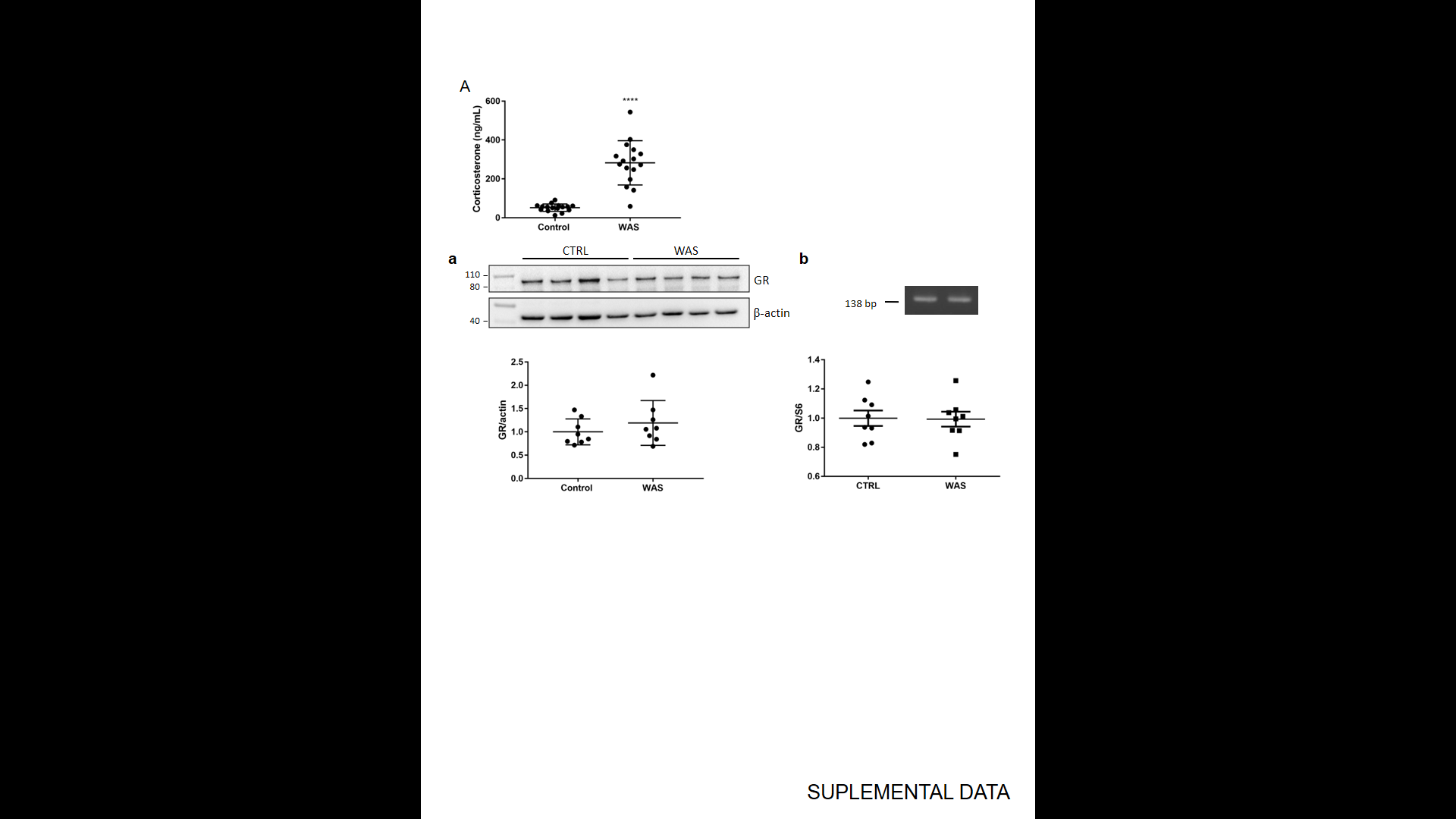


**c**


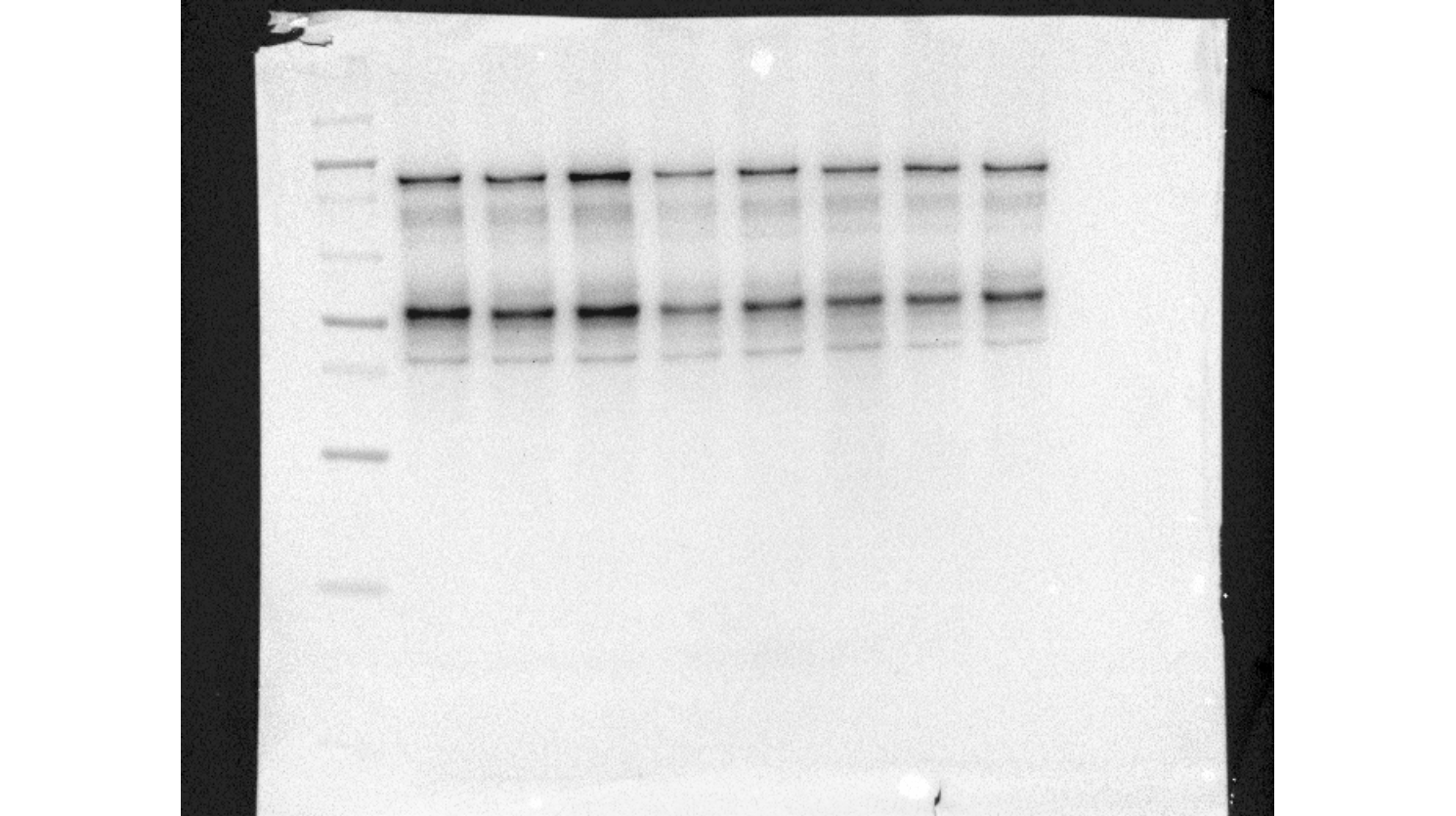


**d**


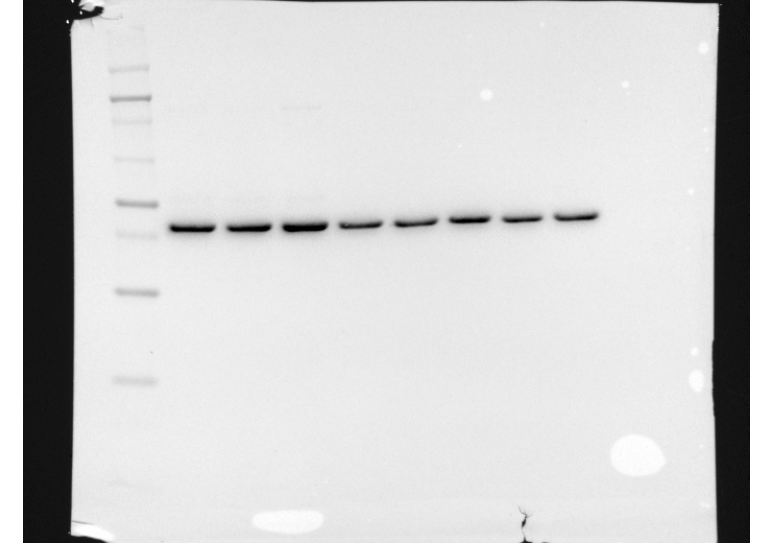


**e**


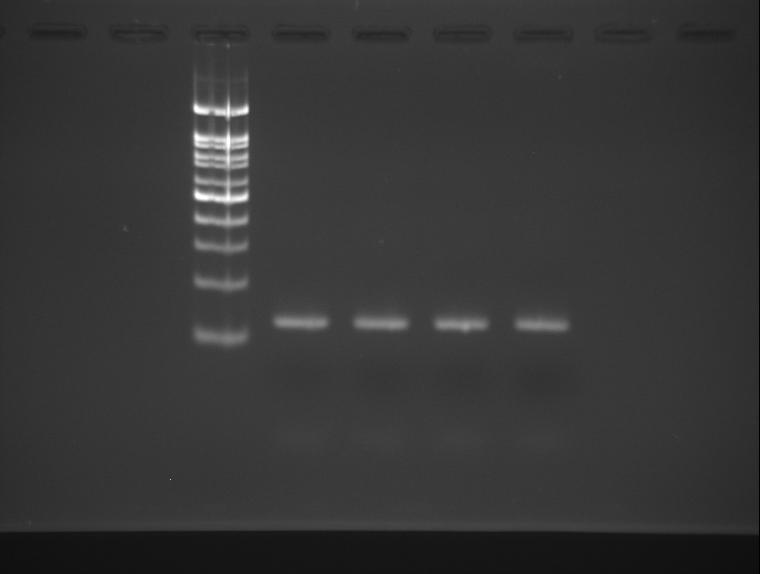


**Supplemental data S4 - RASt did not modify the basal tone of distal colon but increased the area under the curve (AUC) of contractions under basal conditions**

(a) Spontaneous contraction amplitude analysis of distal colon longitudinal muscle segments, without electrical stimulation, under basal condition, after L-Name treatment and after L-Name and Atropine treatment. (b) Amplitude of atropine-sensitive contraction amplitude in control and in WAS mice (n= 8/ group) (c) Analysis of the mean basal tension of distal colon longitudinal muscle segments under basal condition, after L-Name treatment and after L-Name and Atropine treatment (n= 8/ group) (d) Amplitude of atropine-sensitive mean basal tension in control and in WAS mice (n= 8/ group) (e) AUC of spontaneous contractions of distal colon longitudinal muscle segment under basal condition, after L-Name treatment and after L-Name and Atropine treatment (n= 8/ group). (f) Amplitude of atropine-sensitive AUC of spontaneous contractions in control and in WAS mice (n= 8/ group). Data are represented using bars as means +/- SD *p < 0.05, ***p<0.001 Mann-Whitney U test were used for all figures.


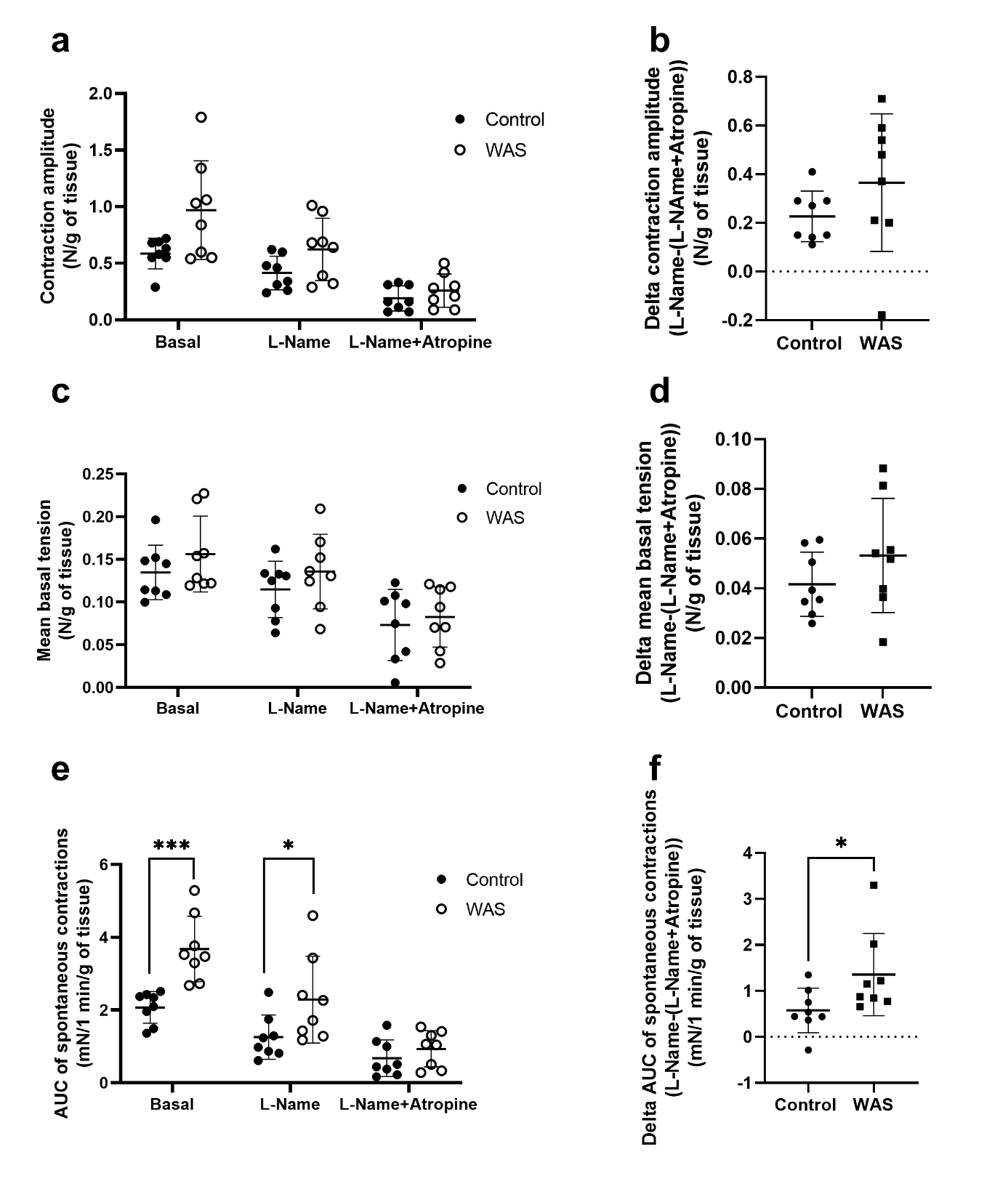

Supplement: Supplementary file 1 [file Data_Sheet_1.docx]
